# Excitatory purinergic and cholinergic expression changed in a partial bladder outlet obstruction-induced overactive bladder rat model

**DOI:** 10.1038/s41598-023-45014-5

**Published:** 2023-10-26

**Authors:** Jingyi Huang, Hongliang Li, Yao Zhang, Jiaye Liu, Hongying Cao, Yongling Long

**Affiliations:** Guangzhou University of Chinese Medicine, No.232 Outer Ring East Road, Higher Education Mega Center, Guangzhou, Guangdong China

**Keywords:** Neurology, Pathogenesis, Urology

## Abstract

Overactive bladder (OAB) is a common, long-term symptom complex with a high prevalence in women worldwide. OAB has caused a social burden, and effective treatments are urgently needed. However, the pathogenesis of OAB has yet to be elucidated. Model rats underwent bladder outlet obstruction surgery. In the 2nd, 3rd, and 4th weeks after surgery, metabolic cages were used to detect the 12 h urine volume of rats in the sham and model groups. The urodynamic parameters bladder leak point pressure (BPLL), maximum voiding pressure (MVP), residual volume (RV), maximum bladder capacity (MBC), bladder compliance (BC), voided efficiency (VE), and non-voiding contractions (NVCs) were also detected. Moreover, the contractile responses of isolated detrusor muscles to electrical and carbachol stimulation were examined at the abovementioned time points. At the 4th week after surgery, the bladders of both groups were obtained for hematoxylin–eosin (H&E) and Masson’s trichrome staining. Real-time qPCR and Western blot were performed to quantify the expression of choline acetyltransferase (ChAT) and solute carrier family 17 member 9 (SLC17A9). At week 4, compared with the sham group, the 12 h urine volume of PBOO group increased significantly. The BLPP, MVP, VE, MBC, and NVCs increased significantly, and the VE was significantly reduced in 4-week PBOO group. The contractile responses of isolated detrusor muscles to electrical and carbachol stimulation significantly increased in 4-week PBOO group. In the 4-week PBOO group, the bladder wall and the ratio of bladder muscle to collagen within the bladder smooth muscle layer wall were significantly higher than those in the sham group. ChAT and SLC17A9 mRNA and protein expression in the OAB model rats significantly increased. At 4 weeks after PBOO, the OAB model was successfully established. The gene and protein expression levels of ChAT and SLC17A9 increased in the bladder of the OAB model, suggesting that OAB may be related to increased excitatory purinergic and cholinergic expression.

## Introduction

Overactive bladder (OAB) is a common lower urinary tract symptom, which was defined by the International Continence Society as the syndrome of urinary urgency, usually accompanied with frequency and nocturia, with or without urgency urinary incontinence (UUI), and in the absence of urinary tract infection or other obvious pathology^1^; it has a high prevalence rate ranging from 11.8% to 17% in women worldwide^2^. The pathophysiological mechanism of OAB remains unclear, and several hypotheses for the pathogenesis of OAB have been proposed, including myogenic, urotheliogenic, urethrogenic, and neurogenic hypotheses^3^.

Cholinergic nerves are nerve fibers that release acetylcholine (Ach) from their endings as a chemical transmitter. The main source of bladder Ach is the parasympathetic nerve, followed by non-neuronal cells of the urothelium. Ach can act on cholinergic receptors in the detrusor, afferent nerve, and urothelium. Bladder contractions are primarily controlled by the cholinergic nerve, followed by purinergic adenosine triphosphate (ATP)-mediated contractions^4^. The parasympathetic cholinergic nerve secretes Ach and binds to the cholinergic receptors on the detrusor muscle, causing the detrusor muscle to contract and promote urination. There are five subtypes of M receptors. In the human bladder, M2 receptors account for about 70% and M3 about 20%. Although M2 is more distributed in the detrusor muscle than M3, the M3 receptor plays a major role in the contraction of the detrusor muscle after activation. In the detrusor, stimulation of the M3 receptors activates phosphoinositide hydrolysis leading to inositol triphosphate (IP3) and diacylglycerol (DAG) formation, which causes the release of calcium from intracellular stores and the influx of extracellular calcium, respectively. Choline acetyltransferase (CHAT), a hallmark enzyme of the cholinergic nerve, can transfer acetyl-CoA to choline to synthesize Ach.

Ach is mainly released from the cholinergic (parasympathetic) efferent nerve. Urothelial non-neuronal Ach can act on M receptors of the detrusor, resulting in myogenic spontaneous contraction. Ach released from the urothelium can also activate urothelial cholinergic receptors and then increase the release of urothelial ATP, thereby increasing afferent activity and producing urgent urination symptoms. Moreover, Ach released from the urothelium can activate afferent nerves, directly contributing to OAB and DO^5,6^. In general, myogenic spontaneous contractions can be caused by increased Ach release from non-neurons and damaged neurons. A previous study demonstrated that the release of Ach from bladder neurons and non-neurons increase in OAB and DO^7^.

In a normal physiological state, non-adrenaline non-cholinergic (NANC) nerve conduction has a synergistic effect with adrenergic and cholinergic nerve conduction, but the proportion is small. For example, human bladder purinergic nerve conduction accounts for only 3% of parasympathetic nerve conduction. However, under pathological conditions, such as DO and OAB, NANC accounts for 40–50%^8^ of bladder nerve conduction, and NANC is an important pathogenesis of many diseases.

The purinergic nerve is an important component of NANC. ATP is a major chemical transmitter in purinergic signal transmission. During the urination phase, purinergic neurotransmitter ATP released by the parasympathetic nerve acts on the detrusor P2X receptor to mediate bladder contraction; in normal bladder physiology, the effect is minor. Bladder smooth muscle of the diabetic bladder dysfunction model in the OAB phase exhibits an increased response to EFS, in which the purinergic component is dominant (about 49–84%), the cholinergic component decreases, and the remaining components of NANC, nitrogenergic innervation, and peptidergic innervation significantly increase^9^. Moreover, in idiopathic detrusor overactivity, the purinergic component increases by about 50%^10^. The increased purinergic conduction may contribute to OAB.

The urothelium releases ATP. During bladder filling, ATP is released from umbrella cells, acting on P2X and P2Y receptors to initiate afferent activity, thereby mediating the sensation of bladder filling and urgency. The increased release of ATP from the urothelium is associated with increased myogenic spontaneous activity. ATP released from the urothelium is increased in patients with OAB and DO^11,12^. Furthermore, both animal and clinical experiments have shown that the parasympathetic nerve releases less Ach and more ATP in the elderly bladder, which may be an important cause of OAB in the elderly; purinergic receptor antagonists may provide potential therapeutic targets for elderly patients with OAB^11,13–15^.

Before secretion, ATP is stored in secretory vesicles found in purinergic cells. Vesicular nucleotide transporter (VNUT) family 17, member 9 (SLC17A9) is an anion transporter family member that can carry nucleotides^16^, such as ATP, adenosine diphosphate (ADP), and guanosine triphosphate (GTP). SlC17A9 can specifically recognize purinergic vesicles of neuronal swelling, is responsible for ATP exocytosis, and is widely expressed in various human and mouse tissues^14^. SLC17A9 has been shown to take up glutamate or ATP into synaptic vesicles or secretory vesicles using the membrane potential driven by V-ATPase. Similar to synaptic vesicles and secretory vesicles, V-ATPase in the lysosome membranes generates substantial voltage potential and an H gradient^17^. SLC17A9 was discovered in various invertebrates and vertebrates, indicating that the molecular mechanisms of purinergic transmission are common in animals^18^.

At present, the most widely used OAB model is the partial bladder outlet obstruction (PBOO) model^19^. Female Sprague–Dawley (SD) rats are the most commonly used animals in the PBOO model^20,21^. The bladder undergoes three sequential stages in PBOO: hypertrophic, compensatory, and decompensated phases^22,23^. The OAB model can be obtained by achieving a compensatory phase after the establishment of PBOO^24^. The OAB rat models are commonly assessed 2, 3, or 4 weeks after PBOO surgery^20,25–27^. In this study, we obtained an appropriate rat model by investigating the changes in bladder and detrusor function in PBOO rat models at different time points. An appropriate rat model was used to further study the gene and protein levels of ChAT and SLC17A9 to clarify the change in excitatory nerve expression in a partial BOO-induced OAB rat model and OAB model to investigate the pathogenesis of OAB.

## Methods

### Ethics statement

All animals were kept in a pathogen-free environment and fed ad libitum. The procedures for care and use of animals were approved by the Ethics Committee of the Guangzhou University of Chinese Medicine (NO.20210125001). The rats were handled according to internationally accepted principles for the care and welfare of laboratory animals, and the experiment was performed following the ARRIVE guidelines (PLoS Bio 8(6), e1000412, 2010). All animals were euthanized in accordance with the American Veterinary Medical Association Guidelines.

### Animal grouping and surgical procedures

**Figure 1:**
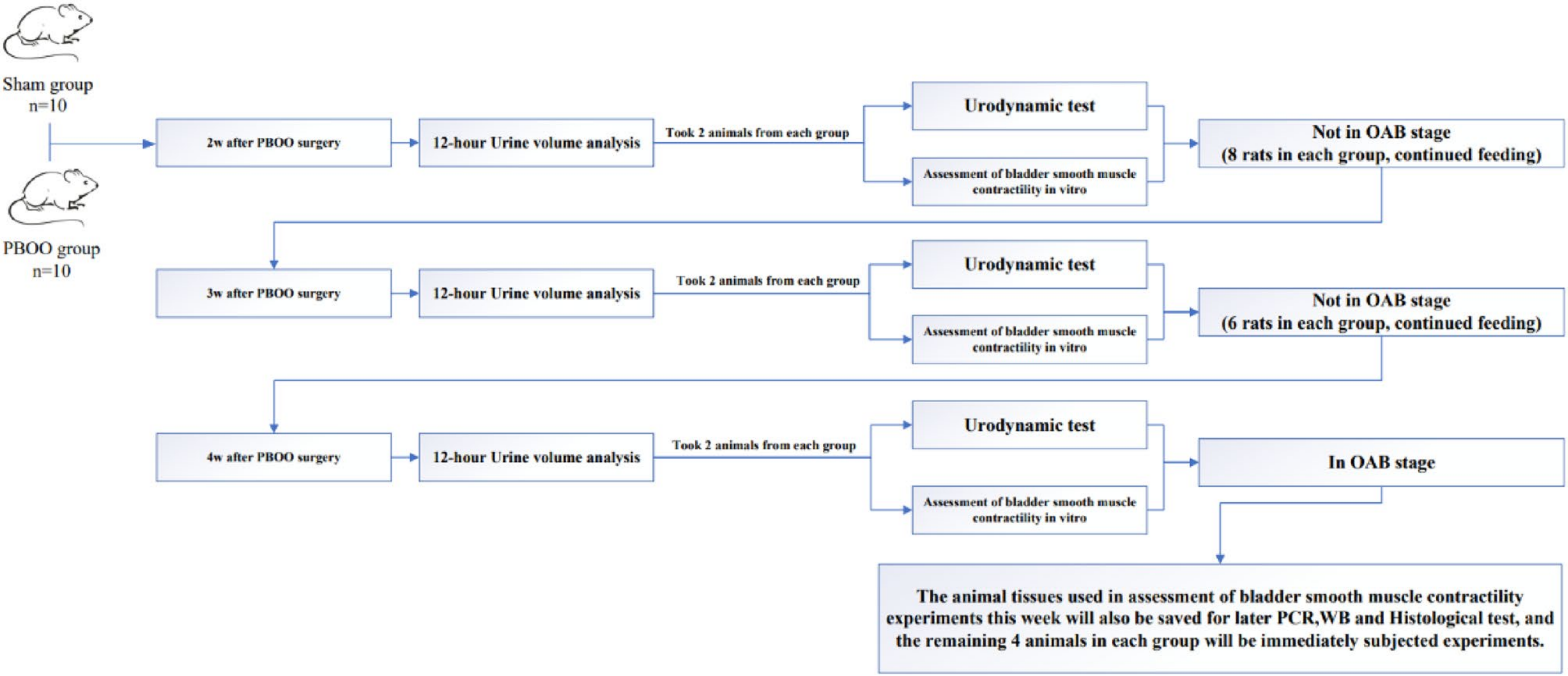
Flow chart of experimental design.

The SD rats (190 ± 20 g) were randomly divided into two groups, namely, the sham-operated and PBOO-operated groups, with 10 animals per group. The PBOO-operated rats were anesthetized with 4% isoflurane and maintained with 2% isoflurane. A 1 mm-diameter polyethylene pipe was gently inserted into the urethra. The bladder was exposed via a 1 cm lower abdominal middle incision. Adipose tissue was removed by blunt dissection to expose the proximal urethra^28^. Using a 3–0 silk ligature, urethra was tied in the presence of a 1 mm polyethylene pipe, which was withdrawn after the tie^29–31^. Sham-operated animals received the same surgical steps without tying the urethra. All the animals received appropriate antibiotics after sutures. The experimental design is shown in Fig. 1.

### 12 Hour urine volume analysis

Before 12 h urine volume analysis, all the animals were housed in metabolic cages individually for 12 h and fasted with ad libitum access to drinking water. After adaptive feeding, the animals continued to be kept in metabolic cages for 12 h, fasting but drinking freely during the experiment, and urine output was measured. At 2, 3, and 4 weeks after PBOO surgery, the 12 h urine volume of the sham and PBOO groups was measured at each time point.

### Urodynamic test

A urodynamic measuring machine was used for urodynamic assessment (Laborite Delphis 94-R01-BT, Canada). Rats were placed in a supine position after anesthesia with 20% uratan (1 g/kg, intraperitoneal injection). Through the urethra, the bladder was then inserted into a 1 mm polyethylene pipe, which was connected to a pressure transducer and a syringe pump via a three-way cutoff valve. The bladder pressure was measured by a pressure transducer and urodynamic measuring machine. Following bladder emptying, the bladder was filled with 0.1 mL/min saline through a syringe pump. During perfusion, the external urethral orifice was watched for liquid overflow, and the bladder pressure at the first drop of liquid overflow was the bladder leak point pressure (BLPP). The bladder pressure curve was observed, and its peak value was recorded, which was the maximum voiding pressure (MVP). The volume of fluid spilled was recorded, which was the void volume (Vv). The maximum bladder capacity (MBC) was determined by the volume of saline injected, which was recorded with a syringe pump. Residual volume (RV) was determined as the urine volume remaining in the bladder after urination and computed using the formula (RV = MBC − Vv.). Void efficiency (VE) was calculated by a formula (VE = Vv / MBC × 100%). The formula (BC = MBC/BLPP × 100%) was used to compute bladder compliance (BC). During the filling phase, the number of contractions that were determined to be non-voiding contractions (NVCs) was counted. The values corresponded to the averages of two voiding cycles for each rat.

### Assessment of bladder smooth muscle contractility in vitro

Rats were euthanized by inhaling carbon dioxide. After euthanasia, the bladder was rapidly removed and inserted into Kreb’s solution. The weight was also determined. The formula of Kreb’s solution is shown in Table 1. The bladder was cut into 2 × 6 lengthwise strips and moved into the Kreb’s solution bath, which was inflated with a mixture of 95% O_2_ and 5% CO_2_ at 37 °C. The ends of the strips were attached to the force sensor, which was connected to a PowerLab (AD Instruments Pty. Ltd., Australia). Before the trials, the strips were balanced under 1 g of passive tension for 1 h. The strips were given EFS (2–64 Hz) stimulation. The strips were then washed and balanced for 45 min. By adding carbachol (CCH; 10–8 M to 10–5 M) to the bath, a cumulative concentration–response curve to CCH was formed. To normalize force data, we measured the weight and length of detrusor strips.

**Table 1:** Formula of Kreb’s solution.

Element	Dosage (mM/L)
NaCl	118
KCL	4.75
MgSO_4_	1.18
NaHCO_3_	24.8
KH_2_PO_4_	1.18
CaCl_2_	2.5
C_6_H_12_O_6_·H_2_O	10

### Real-time PCR

On day 28 following PBOO operation, the animals were sacrificed and the bladders were removed guaranteeing the bladder weight. The bladder was sliced into longitudinal detrusor strips and divided into three sections for RT-qPCR, Western blot, and histological test. RT-qPCR was used to quantify the expression of SLC17A9 and ChAT. The methods were conducted as described in a previous article^32^, using an EZ-press RNA Purification Kit, Color Reverse Transcription Kit, and 2*Color SYBR Green qPCR Master Mix (ROX2 plus), from EZBioscience, USA. Table 2 contains a list of primer pairs. Results were standardized using the expression of GAPDH.

**Table 2:** Primers used for RT-qPCR.

Gene	Primers (5′–3′)
SLC17A9—F	GCTTCCTCAAGGCTATGATCTT
SLC17A9—R	AGGTCCTGAATGTTGACTGAAA
ChAT-F	TGGCCATACCCAGGACACA
ChAT-R	TCCAAGACAAAGAACTGGTTGCA
GAPDH-F	TGAGCATCTCCCTCACAATTCC
GAPDH-R	TTTTTGAGGGTGCAGCGAAC

### Western blot

Western blot was performed to quantify the expression of SLC17A9 and ChAT. The methods were as described in the previous article. The membranes were incubated overnight at 4 °C with rabbit anti-SLC17A9 (1:1000; MBL), sleep anti-ChAT (1:1000; Abcam), and rabbit anti-actin (1:1000; Abcam; 1:1000; CST). Goat anti-rabbit secondary antibody (1:5000, BOSTER) and Donkey anti-goat secondary antibody (1:2000, Servicebio) in 5% non-fat milk were used to incubate for 1 h at room temperature.

### Histological test

The bladder stored at 4% paraformaldehyde solution was embedded in paraffin and sectioned at 5 µm. The sections were stained with hematoxylin and eosin (H&E) and Masson’s trichrome and examined by light microscopy. The bladder wall thickness was determined based on H&E images. The thickness of the bladder wall was measured by graphical analysis software at the same magnification. The ratio of smooth muscle to collagen within the bladder smooth muscle layer wall was measured by using Masson’s trichrome-stained images. Muscles were dyed red, and collagen was dyed blue. The ratio of smooth muscle to collagen within the bladder smooth muscle layer wall was calculated as smooth muscle area/collagen area. The abovementioned parameters were assessed by image analysis software (Image-Pro Plus. 6.0).

### Statistical analysis

Data were expressed as mean values ± standard error of the mean (SEM) analyzed with SPSS 20.0 software. The Kolmogorov–Smirnov test was used to determine the sample distribution’s normality. Student’s t-test was used to compare two groups of independent samples in the case of normality. Nonparametric Mann–Whitney U was used for data that did not conform to normality. ^#^*P* < 0.05, ^##^*P* < 0.01, and ^####^*P* < 0.001 were used for comparisons between the PBOO group and corresponding sham group.

## Result

### Dynamic observation of 12 h urine volume

**Figure 2:**
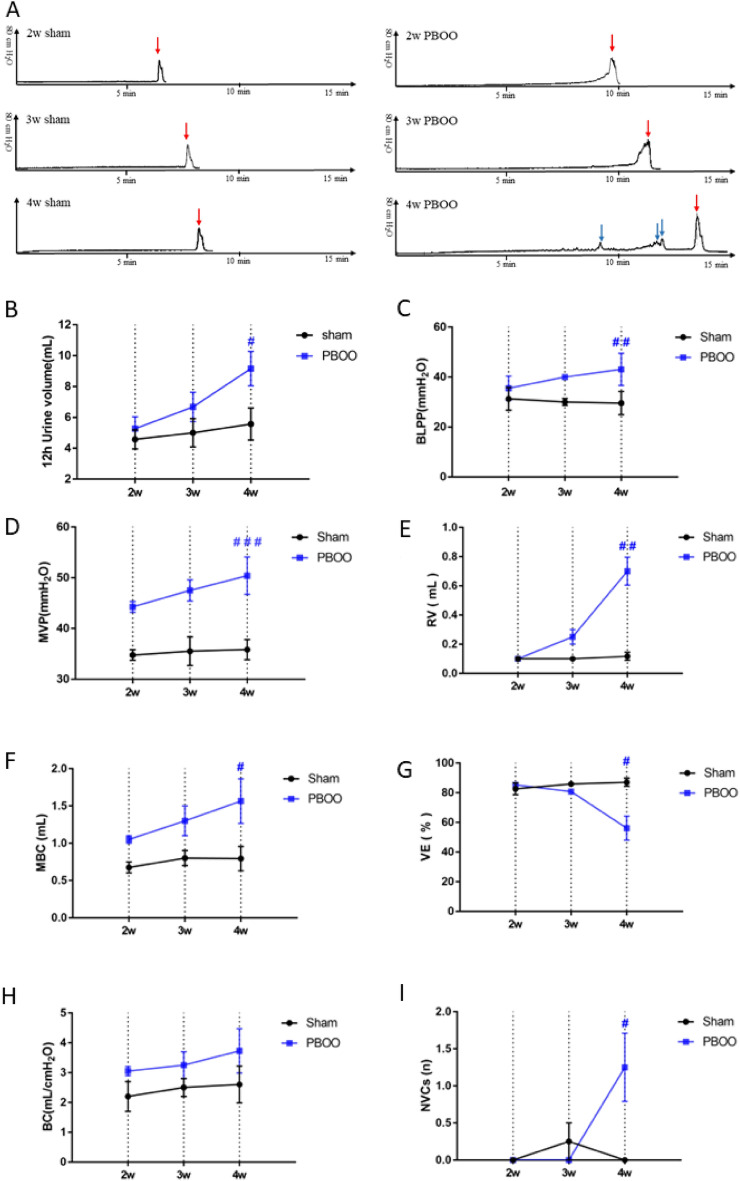
Urine volume (12 h) and urodynamic variables of the sham and PBOO groups were measured at 2, 3, and 4 weeks. (**A**) Typical original record of the urodynamic test performed on the rats in the six groups. (The abscissa is the time, and the ordinate is the pressure. The red arrow marks urination, and the blue arrow marks the non-voiding contraction.) (**B**) Changes in 12 h urine volume, (**C**) bladder leak point pressure (BPLL), (**D**) maximum voiding pressure (MVP), (**E**) residual volume, (RV), (**F**) maximum bladder capacity (MBC), (**G**) voided efficiency (VE), (H) bladder compliance (BC), and (**I**) non-voiding contractions (NVCs). Values are expressed as mean ± SEM. ^#^*P* < 0.05, ^##^*P* < 0.01, and ^####^*P* < 0.001 indicate comparisons between the PBOO group and corresponding sham group.

At 2, 3, and 4 weeks after PBOO surgery, the 12 h urine volume of the sham and PBOO groups was measured (Fig. 2B). The 12 h urine volume of the PBOO group increased gradually at 2, 3, and 4 weeks. Compared with the 4-week sham group, the 12 h urine volume of the 4-week PBOO group significantly increased (*p* < 0.05).

### Dynamic observation of urodynamic parameters

BLPP, MVP, RV, MBC, VE, BC, and NVCs are shown in Fig. 2. Compared with the 2-week sham group, the 2-week PBOO group showed an increase in BLPP, MVP, MBC, and BC but without residual urine and NVCs and a decrease in voided efficiency (VE). BPLL, MVP, MBC, and BC increased sequentially in the 3-week PBOO group and showed slight residual urine and a slight decrease in VE compared with the 3-week sham group.

NVCs were first observed in the 4-week PBOO group. Compared with the 4-week sham group, BPLL, MVP, RV, and MBC significantly increased in the 4-week PBOO group (*P* < 0.01, *P* < 0.001, *P* < 0.01, and *P* < 0.05, respectively), whereas VE significantly decreased (*P* < 0.05). The 4-week PBOO group exhibited an increase in BC, but the difference was not statistically different.

### Dynamic observation of the effect of the stimulus on detrusor contraction

**Figure 3:**
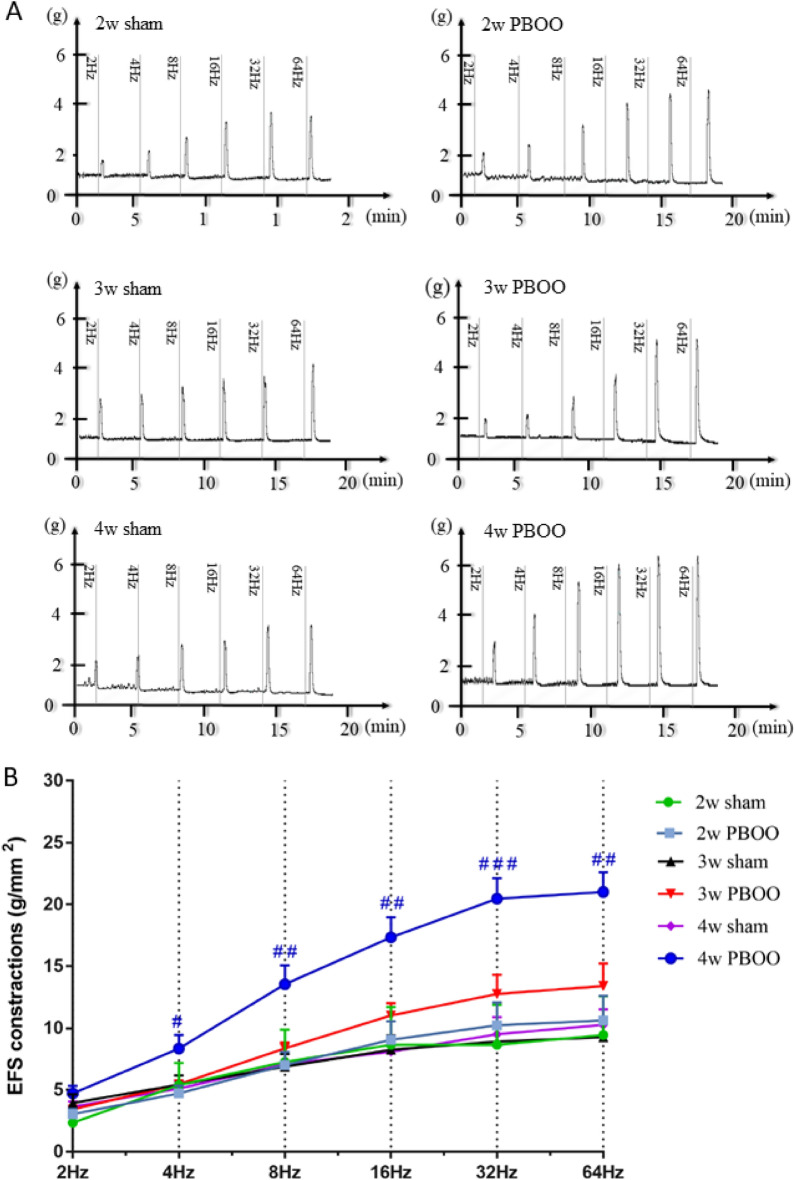
Effect of EFS (2–64 Hz) stimulation on detrusor in vitro was conducted at 2, 3, and 4 weeks. (**A**) Representative original recording of the contraction of detrusor to EFS. (**B**) Detrusor contractile response to EFS from six group rats. Values are expressed as mean ± SEM. ^#^*P* < 0.05, ^##^*P* < 0.01, and ^####^*P* < 0.001 indicate comparisons between the PBOO group and corresponding sham group.

**Figure 4:**
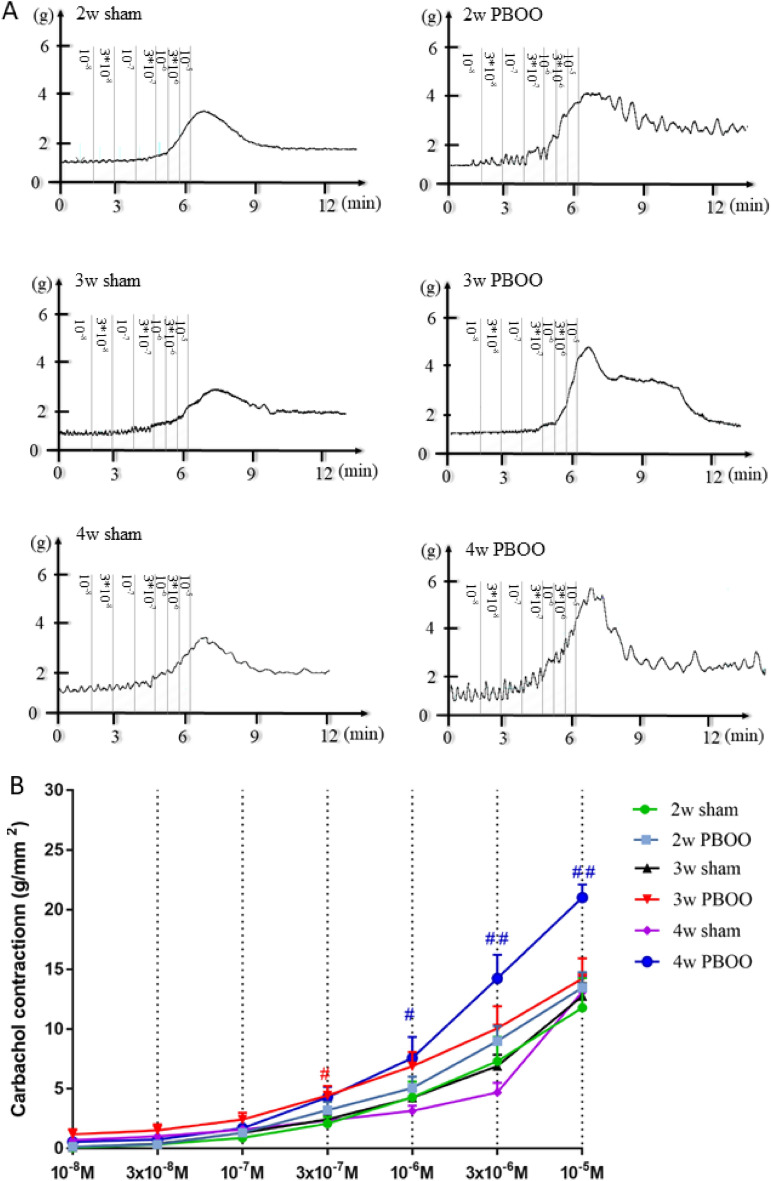
Effect of carbachol (CCH; 10^–8^–10^–5^) stimulation on detrusor in vitro was determined at 2, 3, and 4 weeks. (CCH at concentrations of 10^–8^, 3 × 10^–8^, 10^–7^, and 3 × 10^-7^ M was sequentially added to the bath at 60 s intervals, whereas CCH at concentrations of 10^–6^, 3 × 10^–6^, and 10^-5^ M was added at 30 s intervals.) (**A**) Representative original recording of the contraction of detrusor to CCH (10^–8^–10^–5^). (**B**) Detrusor contractile response to CCH (10^–8^–10^–5^) from six group rats. Values are expressed as mean ± SEM. ^#^*P* < 0.05, ^##^*P* < 0.01, and ^####^*P* < 0.001 indicate comparisons between the PBOO group and corresponding sham group.

At 2, 3, and 4 weeks after PBOO, the effect of EFS (2–64 Hz) and CCH (10^–8^–10^–5^) stimulation on detrusor in vitro was determined (Figs. 3 and 4). The contraction of the detrusor muscle to EFS and CCH stimulation in the PBOO group increased gradually at 2, 3, and 4 weeks after PBOO. Contractions to EFS stimulation were greater in the PBOO group than in the sham group, and statistical differences were observed at week 4 (*P* < 0.05). The response of the detrusor to CCH at 3 × 10^–7^ concentration in the 3-week PBOO group was significantly higher than that in the corresponding sham group. At 4 weeks, the PBOO group had significantly stronger detrusor contraction to CCH (10^–6^–10^–5^) than the 4-week sham group (*P* < 0.05).

### Histological test

**Figure 5:**
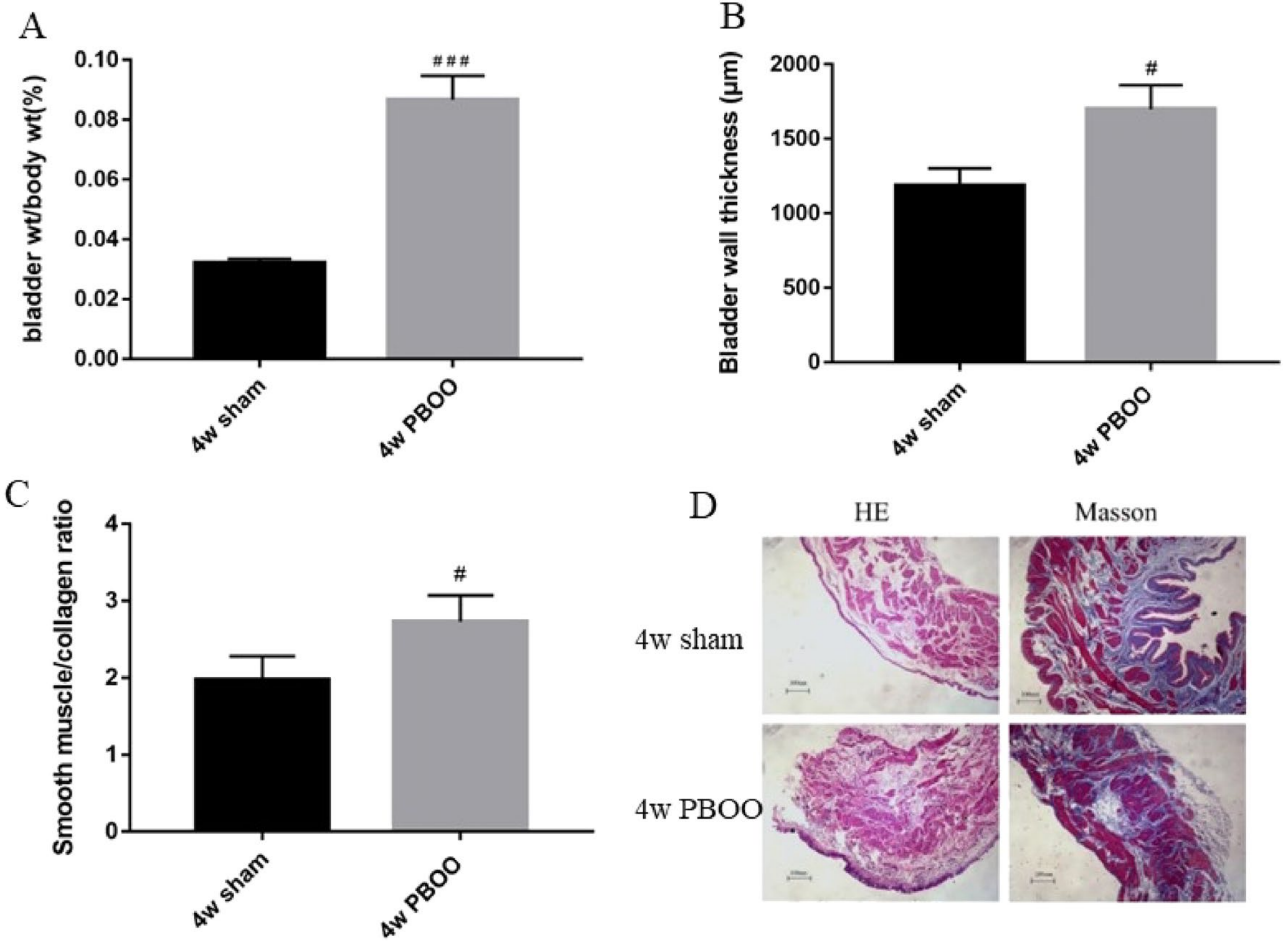
Changes in bladder structure. (**A**) Bladder coefficient was calculated as bladder weight/body weight. (**B**) Bladder wall thickness. (**C**) The ratio of smooth muscle to collagen. (**D**) Representative digitalization images (100 ×) from H&E staining and Masson’s trichrome staining from two groups. Values are expressed as mean ± SEM. ^#^*P* < 0.05, ^##^*P* < 0.01, and ^####^*P* < 0.001 indicate comparisons between the 4-week PBOO group and 4-week sham group.

Bladder coefficient was calculated as bladder weight/body weight. The bladder coefficient of the 4-week PBOO group was substantially higher than that of the 4-week sham group (*P* < 0.001). The bladder wall was measured by H&E staining, and the ratio of smooth muscle to collagen in the smooth muscle layer detected by Masson’s trichrome in the 4-week PBOO group was significantly higher than that in the 4-week sham group (*P* < 0.05; Fig. 5).

### Gene and protein expression levels of ChAT and SLC17A9

**Figure 6:**
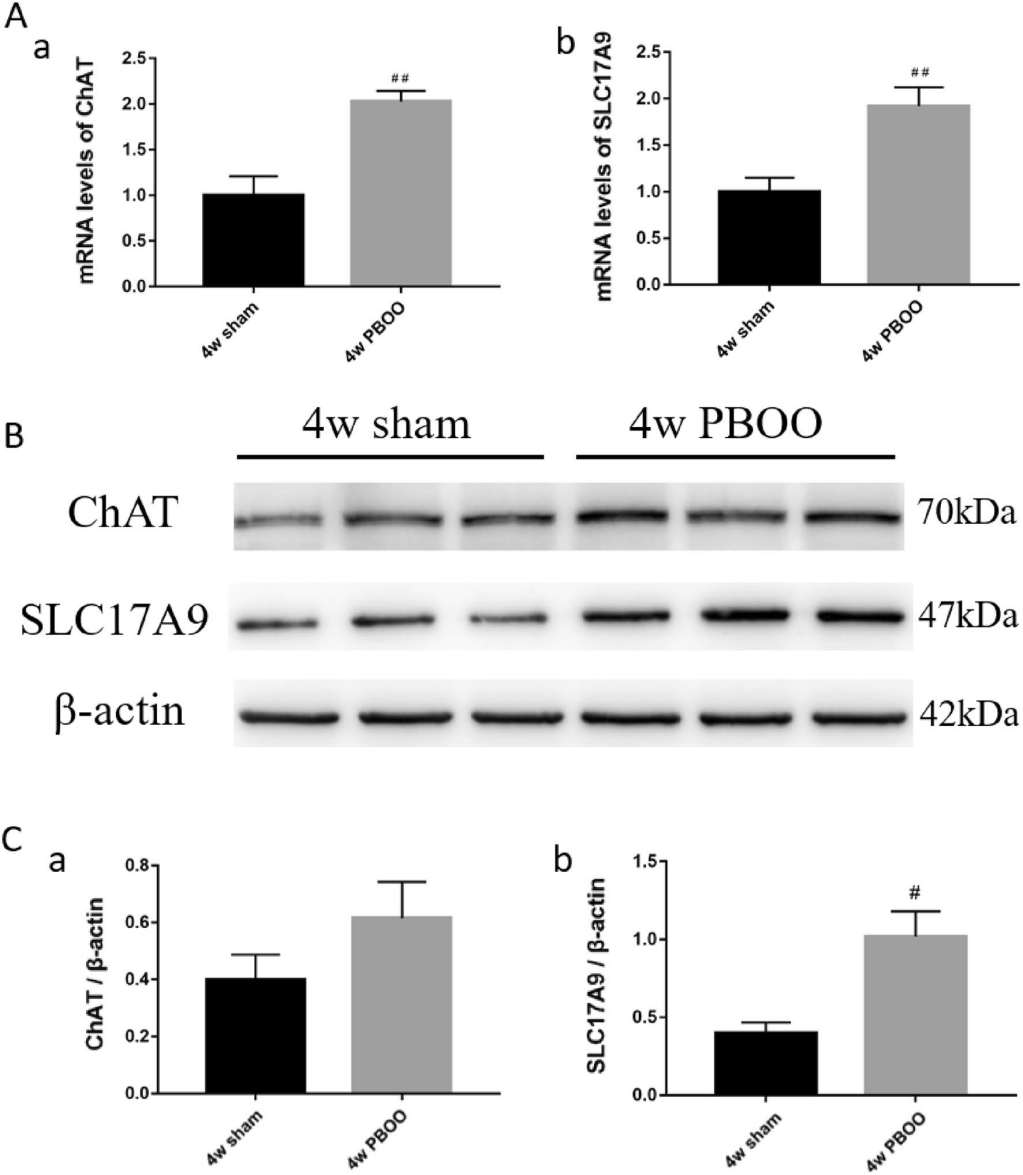
Expression levels of ChAT and SLC17A9 in OAB rat bladder. (**A**) mRNA levels of ChAT (a) and SLC17A9 (b). (**B**) Immunoblots of the protein expression levels of ChAT and SLC17A9. (C) Quantification of protein expression of ChAT (a) and SLC17A9 (b) by normalizing with β-actin. Values are expressed as mean ± SEM. ^#^*P* < 0.05, ^##^*P* < 0.01, and ^####^*P* < 0.001 indicate comparisons between the 4-week PBOO group and 4-week sham group.

Compared with the 4-week sham group, the mRNA expression levels of ChAT and SLC17A9 considerably increased (*P* < 0.01; Fig. 6a). ChAT and SLC17A9 protein expression were higher in the 4-week PBOO group than in the 4-week sham group (Fig. 6c).

## Discussion

Various factors can affect the time to OAB phases after PBOO, namely, species, weight, gender, and level of obstruction. Therefore, to detect the OAB phases, experimental studies are necessary to analyze the changes in bladder and detrusor function in PBOO rat models at 2, 3, and 4 weeks after surgery.

At the early stage after PBOO surgery, the contractile force and contractile time of the detrusor muscle increase, which causes the energy consumption of the detrusor muscle to increase, finally contributing to the rapid increase in bladder weight^33^. At this phase, the bladder can expand to store more urine and release urine through strong pressure, without leading to detrusor dysfunction. This phase is known as hypertrophy. The urodynamic test results in Fig. 2 showed that the BPLL, MVP, MBC, and BC of the 2-week PBOO group increased compared with those in the 2-week sham group; RV, NVCs, and decrease in VE were not detected in this stage. The urodynamic characteristics of the 2-week PBOO group in this experiment were similar to those in the hypertrophy stage^34^. Besides, the 12 h urine volume in the 2-week PBOO group was higher than that in the 2-week sham group, and the difference was not significant.

Electrical stimulation of isolated detrusor muscles activates the release of neurotransmitters from detrusor nerve terminals and induces frequency-dependent contraction. In normal bladder, this contractile response is mainly caused by Ach activation of cholinergic receptors, another is mediated by ATP activation of purinergic receptors, and about 5% of the contractile response is mediated by NANC components^35^. CCH is a cholinomimetic drug and a non-selective M-receptor agonist. The responses of isolated detrusors to EFS and CCH stimulation can reflect the changes in detrusor function at different stages^36^.

In this study, compared with the 2-week sham group, the contraction of the isolated detrusor to EFS and CCH stimulation in the 2-week PBOO group increased, but the difference was not statistically significant. Combining the results of the urodynamic test, in this stage, detrusor dysfunction had not yet occurred and the bladder outlet obstruction was compensated by expanding the bladder capacity and increasing the voiding pressure, similar to the hypertrophy stage. Therefore, research on the next time point should be continued to achieve the compensated stage.

In the 3-week PBOO group, the BLPP, MVP, MBC, and BC continued to increase with no statistical difference. Compared with the 3-week sham group, the 3-week PBOO group showed less severe RV and a slight decrease in VE; no NVC was observed. Furthermore, the 12 h urine volume of the 3-week PBOO group was higher than that in the 3-week sham group with no statistical difference. However, compared with the 2-week PBOO group, the above parameters continued to show an upward trend. Compared with the 2-week PBOO group, the urodynamic indexes and 12 h urine volume in the 3-week PBOO group showed a trend of further development to the OAB stage.

The response of the isolated detrusor to EFS and CCH stimulation in the 3-week PBOO group increased compared with that in the 2-week PBOO group. Moreover, compared with the 3-week sham group, the response of the isolated detrusor significantly increased under stimulation with CCH at 3 × 10^–7^ concentration. This result suggested that the OAB phase was about to enter after 3 weeks after PBOO surgery, so the observation at 4 weeks was continued.

The compensatory phase occurs after hypertrophy phase, during which the bladder weight becomes stable^37^. However, persistent bladder hypertrophy and persistent increase in detrusor contractility and contraction time in the early stage lead to high energy loss and detrusor dysfunction, resulting in NVCs that can cause frequent urination and urge incontinence. During the compensatory period, urination pressure increases, whereas the bladder emptying capacity decreases, increasing residual urine volume; thus, the next stage reaches the maximum bladder capacity early, shortening the urination interval and increasing the urination frequency^38^.

In this study, compared with the 4-week sham group, BLPP, MVP, MBC, RV, and NVCs in the 4-week PBOO group increased significantly, and VE was significantly decreased. The 12 h urine volume of the 4-week PBOO group continued to increase, which was statistically different from that of the 4-week sham group. Previous studies reported similar results for the urodynamic parameters of the PBOO-induced OAB models^34,39^.

Furthermore, in the 4-week PBOO group, the responses of the isolated detrusor to EFS and CCH stimulation continued to increase compared with those in the 2-week and 3-week PBOO groups, and they were significantly higher than those in the 4-week sham group. These results indicated that it entered the OAB phase at 4 weeks after the operation, and the OAB model was successfully established^40^.

The isolated detrusor finally enters the decompensated stage after staying in the compensated stage for a while, with bladder weight increasing rapidly^41^. At the end of decompensation, smooth muscle cells decrease, forming a large, thin-walled fibrous bladder or a small, thick-walled fibrous bladder. The micturition pressure decreases because of fibrosis and degeneration bladder. In the decompensated stage, detrusor innervation is impaired, the response to EFS and CCH stimulation is reduced, and the bladder emptying ability is gradually lost, which result in the further increase in residual urine volume^42^.

The present study revealed urodynamic changes in the PBOO rats in the hypertrophy and compensated stages that followed PBOO. The rat model for the OAB study was chosen in week 4 after the PBOO operation. In the OAB model, the bladder coefficient, bladder wall thickness, and the ratio of smooth muscle to collagen within bladder smooth muscle were significantly higher than those in the sham group, similar to the findings reported by a previous study^36,43^. The results of hypertrophy of the bladder are as follows. In the early stage of BOO, the main change in bladder wall is the thickening of the muscle layer caused by the proliferation of detrusor muscle cells^44^. Collagen composition is increased, but the smooth muscle/collagen ratio is increased compared with the proliferation of detrusor muscle^43^. Collagen fibers are sparse, but detrusor fibers are dense. Bladder emptying ability may be related to the bladder structure. On the one hand, the hypertrophic bladder wall increases the contractility of the bladder, which is conducive to the emptying of the bladder. On the other hand, the proliferation of the detrusor muscle and the decrease in the relative composition of collagen may lead to increased detrusor stiffness, which is not conducive to bladder emptying, producing residual urination^45^.

ChAT was used to label cholinergic nerves and study Ach release^46,47^. The results of this study showed that ChAT expression increased in PBOO-induced OAB rat models, suggesting that the release of excitatory neurotransmitters Ach in bladder increased, which may lead to the increase of detrusor autonomic contraction, thereby triggering OAB. Previous studies reported similar results. ChAT inhibitors have been shown to improve OAB symptoms^42,48^. In diabetic rats with bladder overactivity, ChAT expression was increased in bladder tissue and primary bladder neurons^49,50^, which was similar to our findings. Levin RM et al. showed that ChAT activity increases in the early stage of bladder outlet obstruction (OAB stage), and ChAT activity decreases in the later stage (decompensated stage).

Numerous studies have proven that purinergic‐induced contraction significantly increases under OAB^9–12,51^, but whether this increased contraction is caused by the increase in ATP release needs to be studied further. Increased release of ATP from the urothelium of spinal cord injured bladders may contribute to the development of bladder hyperactivity^52^. SLC17A9 can be used to study the release of ATP^53^. In this study, the gene and protein levels of SLC17A9 were higher than those in the sham group, suggesting that the release of excitatory neurotransmitters ATP in bladder increased. Similar results were noted in other OAB models. Increased expression of SLC17A9 protein in the bladder was detected in diabetic bladder dysfunction induced by streptozotocin (STZ) in male C57BL/6 mice. However, during the decompensation phase of diabetic bladder dysfunction, SLC17A9 expression is reduced^53^. KK-Ay is the most common model of spontaneous type 2 diabetes. In KK-ay diabetic dysfunction, SLC17A7 mRNA expression was elevated in the OAB phase. Selective regulation of ATP release may offer a way to treat OAB.

## Conclusions

We established an OAB model by dynamically observing the changes in bladder function and detrusor function after PBOO. The results of OAB model provide the basis for future OAB research. We revealed increased bladder ChAT and SLC17A9 expression in PBOO-induced OAB models, suggesting the increased release of excitatory neurotransmitters Ach and ATP in OAB models. This study provides evidence for the pathogenesis of OAB and includes possible treatment options for the treatment of OAB (Supplementary file).

### Supplementary Information

Supplementary Information.

## Data Availability

Data will be made available on request (please contact 1289574479@qq.com).

## References

[1] Lightner DJ, Gomelsky A, Souter L, Vasavada SP (2019). Diagnosis and treatment of overactive bladder (non-neurogenic) in adults: AUA/SUFU guideline amendment 2019. J. Urol..

[2] Geoffrion R (2018). No. 283-Treatments for overactive bladder: Focus on pharmacotherapy. J. Obstet. Gynaecol. Can..

[3] Peyronnet B, Mironska E, Chapple C, Cardozo L, Oelke M, Dmochowski R, Amarenco G, Gamé X, Kirby R, Van Der Aa F (2019). A comprehensive review of overactive bladder pathophysiology: On the way to tailored treatment. Eur. Urol..

[4] McCarthy CJ, Ikeda Y, Skennerton D, Chakrabarty B, Kanai AJ, Jabr RI, Fry CH (2019). Characterisation of nerve-mediated ATP release from bladder detrusor muscle and its pathological implications. Br. J. Pharmacol..

[5] Andersson KE (2011). Antimuscarinic mechanisms and the overactive detrusor: An update. Eur. Urol..

[6] Ohtake A, Sato S, Sasamata M, Miyata K (2010). The forefront for novel therapeutic agents based on the pathophysiology of lower urinary tract dysfunction: Ameliorative effect of solifenacin succinate (Vesicare), a bladder-selective antimuscarinic agent, on overactive bladder symptoms, especially urgency episodes. J. Pharmacol. Sci..

[7] Sellers DJ, Chess-Williams R (2012). Muscarinic agonists and antagonists: Effects on the urinary bladder. Handb. Exp. Pharmacol..

[8] Kamiyama Y, Muto S, Masuda H, Ide H, Ishizuka N, Saito K, Horie S (2008). Inhibitory effects of nicorandil, a K ATP channel opener and a nitric oxide donor, on overactive bladder in animal models. BJU Int..

[9] Cao N, Gu B, Gotoh D, Yoshimura N (2019). Time-dependent changes of urethral function in diabetes mellitus: A review. Int. Neurourol. J..

[10] Moore KH, Ray FR, Barden JA (2001). Loss of purinergic P2X(3) and P2X(5) receptor innervation in human detrusor from adults with urge incontinence. J. Neurosci..

[11] Chakrabarty B, Ito H, Ximenes M, Nishikawa N, Vahabi B, Kanai AJ, Pickering AE, Drake MJ, Fry CH (2019). Influence of sildenafil on the purinergic components of nerve-mediated and urothelial ATP release from the bladder of normal and spinal cord injured mice. Br. J. Pharmacol..

[12] Firouzmand S, Ajori L, Towse J, Allameh F, Najafi S, Javed S, John B, Langley SEM, Fry CH, Young JS (2020). Investigating the associations of mucosal P2Y6 receptor expression and urinary ATP and ADP concentrations, with symptoms of overactive bladder. Neurourol. Urodyn..

[13] Yoshida M, Miyamae K, Iwashita H, Otani M, Inadome A (2004). Management of detrusor dysfunction in the elderly: Changes in acetylcholine and adenosine triphosphate release during aging. Urology.

[14] Andersson KE (2015). Purinergic signalling in the urinary bladder. Auton. Neurosci..

[15] Firouzmand S, Ajori L, Towse J, Allameh F, Najafi S, Javed S, John BS, Langley SEM, Fry CH, Young JS (2020). Investigating the associations of mucosal P2Y6 receptor expression and urinary ATP and ADP concentrations, with symptoms of overactive bladder. Neurourol. Urodyn..

[16] Nakagomi H, Yoshiyama M, Mochizuki T, Miyamoto T, Komatsu R, Imura Y, Morizawa Y, Hiasa M, Miyaji T, Kira S (2016). Urothelial ATP exocytosis: Regulation of bladder compliance in the urine storage phase. Sci. Rep..

[17] Cao Q, Zhao K, Zhong XZ, Zou Y, Yu H, Huang P, Xu TL, Dong XP (2014). SLC17A9 protein functions as a lysosomal ATP transporter and regulates cell viability. J. Biol. Chem..

[18] Sawada K, Echigo N, Juge N, Miyaji T, Otsuka M, Omote H, Yamamoto A, Moriyama Y (2008). Identification of a vesicular nucleotide transporter. Proc. Natl. Acad. Sci. U S A.

[19] Parsons BA, Drake MJ (2011). Animal models in overactive bladder research. Handb. Exp. Pharmacol..

[20] Park EC, Lim JS, Kim SI, Lee SY, Tak YK, Choi CW, Yun S, Park J, Lee M, Chung HK (2018). Proteomic analysis of urothelium of rats with detrusor overactivity induced by bladder outlet obstruction. Mol. Cell Proteomics.

[21] Kitta T, Kanno Y, Chiba H, Higuchi M, Ouchi M, Togo M, Moriya K, Shinohara N (2018). Benefits and limitations of animal models in partial bladder outlet obstruction for translational research. Int. J. Urol..

[22] Yu ZJ, Yan HL, Xu FH, Chao HC, Deng LH, Xu XD, Huang JB, Zeng T (2020). Efficacy and side effects of drugs commonly used for the treatment of lower urinary tract symptoms associated with benign prostatic hyperplasia. Front. Pharmacol..

[23] Bosch R, Abrams P, Averbeck MA, Finazzi Agró E, Gammie A, Marcelissen T, Solomon E (2019). Do functional changes occur in the bladder due to bladder outlet obstruction?—ICI-RS 2018. Neurourol. Urodyn..

[24] Fusco F, Creta M, De Nunzio C, Iacovelli V, Mangiapia F, Li Marzi V, Finazzi Agrò E (2018). Progressive bladder remodeling due to bladder outlet obstruction: A systematic review of morphological and molecular evidences in humans. BMC Urol..

[25] Kang YJ, Jin LH, Park CS, Shin HY, Yoon SM, Lee T (2011). Early sequential changes in bladder function after partial bladder outlet obstruction in awake sprague-dawley rats: Focus on the decompensated bladder. Korean J. Urol..

[26] Choi JB, Jeon SH, Kwon EB, Bae WJ, Cho HJ, Ha US, Hong SH, Lee JY, Kim SW (2020). The effects of oral administration of the novel muscarinic receptor antagonist DA-8010 on overactive bladder in rat with bladder outlet obstruction. BMC Urol..

[27] Zhou F, Li H, Zhou C, Lv H, Ma Y, Wang Y, Song BO (2016). Structural and functional changes in gap junctional intercellular communication in a rat model of overactive bladder syndrome induced by partial bladder outlet obstruction. Exp. Ther. Med..

[28] Wang Y, Xiong Z, Gong W, Zhou Z, Lu G (2015). Urethral orifice hyaluronic acid injections: A novel animal model of bladder outlet obstruction. BMC Urol..

[29] Abramowitch SD, Feola A, Jallah Z, Moalli PA (2009). Tissue mechanics, animal models, and pelvic organ prolapse: A review. Eur. J. Obstet. Gynecol. Reprod. Biol..

[30] Chen L, Lv L, Zhang L, Gao Z, Liu Y, Wang S, Zhou N, Xia Y, Cui J, Jiang X (2021). Metformin ameliorates bladder dysfunction in a rat model of partial bladder outlet obstruction. Am. J. Physiol. Ren. Physiol..

[31] Jin LH, Andersson KE, Han JU, Kwon YH, Park CS, Shin HY, Yoon SM, Lee T (2011). Persistent detrusor overactivity in rats after relief of partial urethral obstruction. Am. J. Physiol. Regul. Integr. Comp. Physiol..

[32] Pan Y, Shu G, Fu L, Huang K, Zhou X, Gui C, Liu H, Jin X, Chen M, Li P (2023). EHBP1L1 drives immune evasion in renal cell carcinoma through binding and stabilizing JAK1. Adv. Sci. (Weinh).

[33] Kanno Y, Mitsui T, Kitta T, Moriya K, Tsukiyama T, Hatakeyama S, Nonomura K (2016). The inflammatory cytokine IL-1β is involved in bladder remodeling after bladder outlet obstruction in mice. Neurourol. Urodyn..

[34] Lai H, Tan B, Liang Z, Yan Q, Lian Q, Wu Q, Huang P, Cao H (2015). Effect of the Chinese traditional prescription Suo Quan Wan on TRPV1 expression in the bladder of rats with bladder outlet obstruction. BMC Complement. Altern. Med..

[35] Lai HH, Smith CP, Munoz A, Boone TB, Szigeti GP, Somogyi GT (2008). Activation of cholinergic receptors blocks non-adrenergic non-cholinergic contractions in the rat urinary bladder. Brain Res. Bull..

[36] Kendig DM, Ets HK, Moreland RS (2016). Effect of type II diabetes on male rat bladder contractility. Am. J. Physiol. Ren. Physiol..

[37] Levin RM, Haugaard N, O'Connor L, Buttyan R, Das A, Dixon JS, Gosling JA (2000). Obstructive response of human bladder to BPH versus rabbit bladder response to partial outlet obstruction: a direct comparison. Neurourol. Urodyn..

[38] Chapple CR, Osman NI (2022). Underactive bladder versus bladder outlet obstruction: Don't Get Tricked!. Eur. Urol. Focus.

[39] Kang HJ, Kim SW, Lee YS, Han SW, Kim JH (2020). Effects of 1% lidocaine instillation on overactive bladder induced by bladder outlet obstruction in rats. Urol. J..

[40] Filippi S, Morelli A, Sandner P, Fibbi B, Mancina R, Marini M, Gacci M, Vignozzi L, Vannelli GB, Carini M (2007). Characterization and functional role of androgen-dependent PDE5 activity in the bladder. Endocrinology.

[41] Matsumoto S, Hanai T, Uemura H, Levin RM (2009). Effects of chronic treatment with vardenafil, a phosphodiesterase 5 inhibitor, on female rat bladder in a partial bladder outlet obstruction model. BJU Int..

[42] Mustafa S, Ismael HN (2014). Reactivity of diabetic urinary bladder to the cholinesterase inhibitor neostigmine. Urology.

[43] Liu Q, Luo D, Yang T, Liao B, Li H, Wang KJ (2017). Protective effects of antimuscarinics on the bladder remodeling after bladder outlet obstruction. Cell Physiol. Biochem..

[44] Yu G, Bo S, Xiyu J, Enqing X (2003). Effect of bladder outlet obstruction on detrusor smooth muscle cell: An in vitro study. J. Surg. Res..

[45] Khan MA, Thompson CS, Angelini GD, Morgan RJ, Mikhailidis DP, Jeremy JY (1999). Prostaglandins and cyclic nucleotides in the urinary bladder of a rabbit model of partial bladder outlet obstruction. Prostaglandins Leukot Essent Fatty Acids.

[46] Bschleipfer T, Weidner W, Kummer W, Lips KS (2012). Does bladder outlet obstruction alter the non-neuronal cholinergic system of the human urothelium?. Life Sci..

[47] Yoshida M, Inadome A, Maeda Y, Satoji Y, Masunaga K, Sugiyama Y, Murakami S (2006). Non-neuronal cholinergic system in human bladder urothelium. Urology.

[48] Nakai M, Akino H, Kaneda T, Matsuta Y, Shiyama R, Tanase K, Ito H, Aoki Y, Oyama N, Miwa Y (2006). Acetylcholinesterase inhibitor acting on the brain improves detrusor overactivity caused by cerebral infarction in rats. Neuroscience.

[49] Wahba ZZ, Soliman KF, Kolta MG (1992). Effect of diabetes on the cholinergic enzyme activities of the urinary bladder and the seminal vesicles of the rat. Exp. Clin. Endocrinol..

[50] Zhang Y, Zhang J, Hong M, Huang J, Xu S, Wang R, Zhou N, Huang P, Tan B, Cao H (2022). Suo Quan Wan ameliorates bladder overactivity and regulates neurotransmission via regulating Myosin Va protein expression. Phytomedicine.

[51] Leiria LO, Mónica FZ, Carvalho FD, Claudino MA, Franco-Penteado CF, Schenka A, Grant AD, De Nucci G, Antunes E (2011). Functional, morphological and molecular characterization of bladder dysfunction in streptozotocin-induced diabetic mice: evidence of a role for L-type voltage-operated Ca^2+^ channels. Br. J. Pharmacol..

[52] Khera M, Somogyi GT, Kiss S, Boone TB, Smith CP (2004). Botulinum toxin A inhibits ATP release from bladder urothelium after chronic spinal cord injury. Neurochem. Int..

[53] Wang J, Lian DW, Yang XF, Xu YF, Chen FJ, Lin WJ, Wang R, Tang LY, Ren WK, Fu LJ (2019). Suo Quan Wan protects mouse from early diabetic bladder dysfunction by mediating motor protein Myosin Va and transporter protein SLC17A9. Front. Pharmacol..

