# Supplementary material for: Excitatory purinergic and cholinergic expression changed in a partial bladder outlet obstruction-induced overactive bladder rat model
Source: Sci Rep. 2023 Oct 26;13:18395. doi: 10.1038/s41598-023-45014-5 (PMC10603080; doi:10.1038/s41598-023-45014-5)
Supplement: Supplementary file 1 — Supplementary Information. [file 41598_2023_45014_MOESM1_ESM.docx]

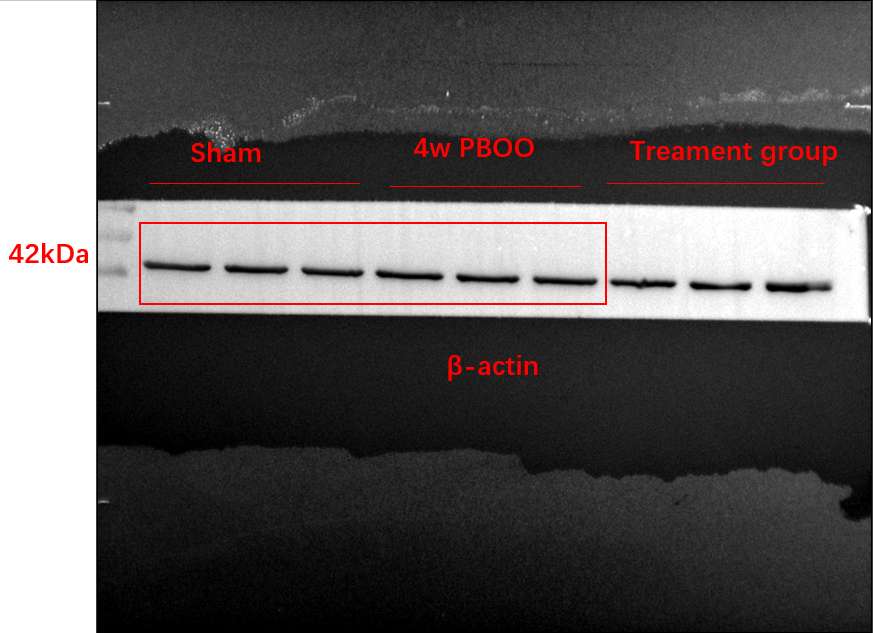

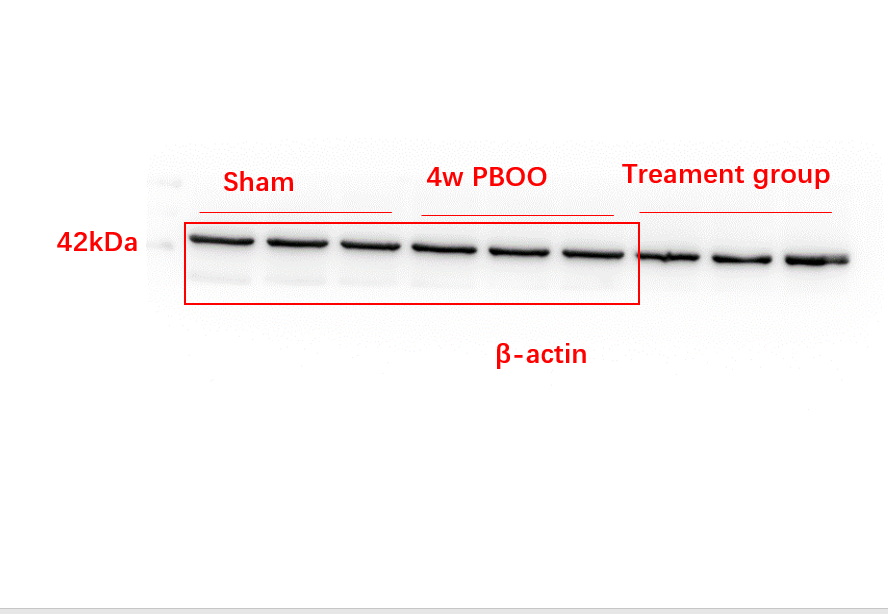


FIG1 The blot of β-actin

(The treatment group is not included in this article, but for the sake of picture integrity, the author has not captured it. The blots were cut prior to hybridisation with antibodies during blotting. )


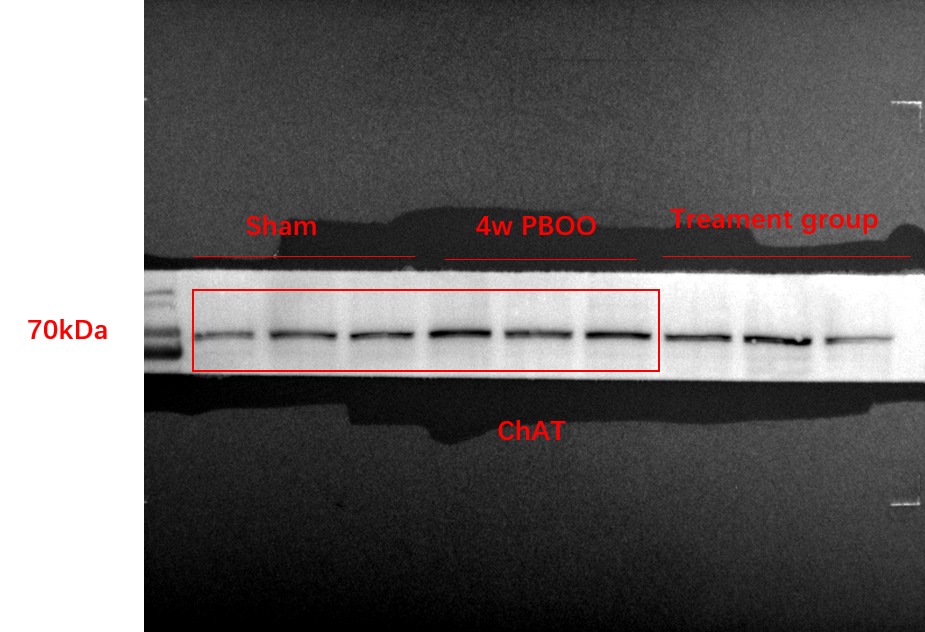

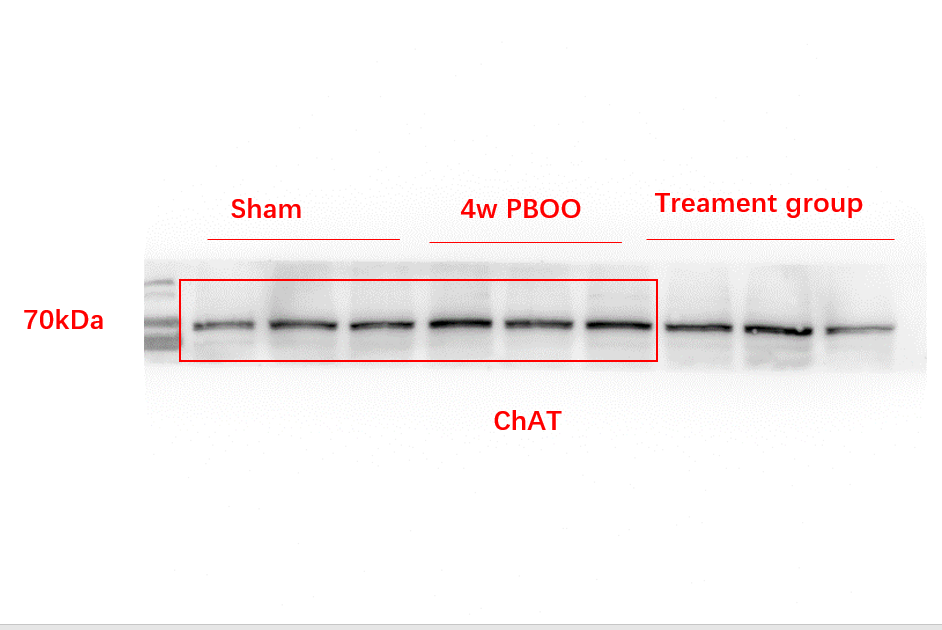


FIG2 The blot of ChAT

(The treatment group is not included in this article, but for the sake of picture integrity, the author has not captured it. The blots were cut prior to hybridisation with antibodies during blotting)


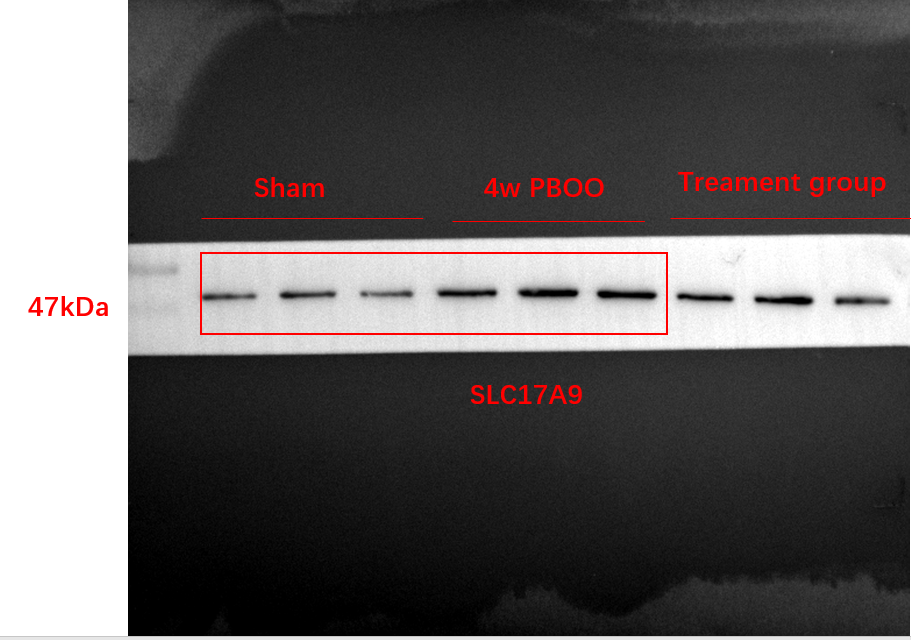

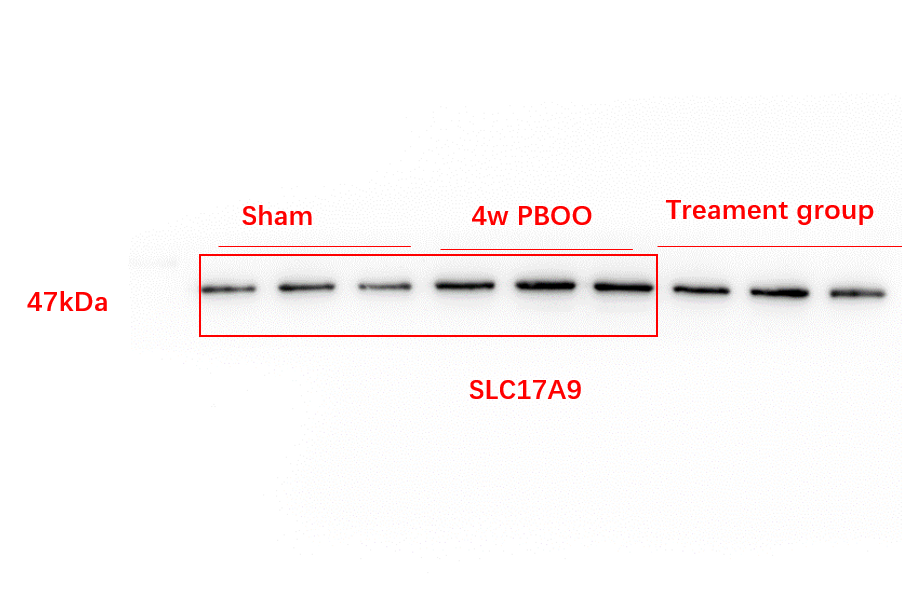


FIG3 The blot of SLC17A9

(The treatment group is not included in this article, but for the sake of picture integrity, the author has not captured it. The blots were cut prior to hybridisation with antibodies during blotting)
